# The Chemistry of Alliums

**DOI:** 10.3390/molecules23010143

**Published:** 2018-01-11

**Authors:** Martin C. H. Gruhlke, Alan J. Slusarenko

**Affiliations:** Department of Plant Physiology, RWTH Aachen University, D-52056 Aachen, Germany; Martin.Gruhlke@rwth-aachen.de

Physiologically active sulfur-containing compounds produced by *Allium* spp. have long fascinated chemists, biochemists, and biologists. The major focus of attention has been on garlic compounds where the starting point for the complex chemistry is alliin (*S*-allylcysteine sulfoxide). Alliin is acted upon by the cysteine-*S*-lyase enzyme alliinase, when cell damage mixes substrate and enzyme. The first major volatile product is allicin (diallylthiosulfinate), giving crushed garlic its characteristic odor. Allicin decomposes readily to allylsulfenic acid (2-propensulfenic acid) and thioacreolin (2-propenethial), which enter into a cascade of reactions producing alkyl disulfides including diallyl disulfide and various polysulfanes, vinyl dithiins, and ajoene.

This special issue, “The Chemistry of Alliums”, contains nine contributions that report on the chemistry and physiology of *Allium* organosulfur compounds. The paper by Eric Block et al. merits particular attention, as it introduces a new facet to the subject by reporting on the synthesis and physiological activity of fluorinated analogues of garlic organosulfur compounds [1]. Continuing the theme of taking garlic substances as lead compounds, Siyo et al., in an elegant molecular biological study, report on the activation of the unfolded protein response as the mechanism of cytotoxicity of the ajoene analogue bisPMB [2].

Garlic is consumed in many forms worldwide, and interest in the potential health benefits of aged, or ‘black garlic’, which no longer contains allicin but is enriched with several downstream metabolites, is increasing. Five contributions in this special issue relate to aged garlic and its sulfur-containing constituents. Ryu and Kang [3] contribute an up-to-date review on the reported physiological activities and constituents of aged black garlic, and Farrag et al. compare the effects of different drying methods in an MS-based metabolomics study [4]. Aged black garlic is enriched in S-allyl cysteine (SAC), and Tsukoa et al. report on the beneficial effects of SAC on pulmonary fibrosis in rats [5]. Kodera et al. report on the biological properties of the closely related S-1-propenyl-l-cysteine [6], and Pérez-Torres et al. report on the effect of the extracts of aged garlic cardiovascular function in rats exhibiting metabolic syndrome [7].

The assimilation mechanisms that plants use for SO_4_^2−^ can also be used for SeO_4_^2−^ or SeO_3_^2−^, and the sulfur-rich alliums are often good dietary sources of Se, which is frequently deficient in the human diet and is very important for antioxidative, protective enzymes, such as glutathione peroxidase. However, cultivated alliums can only synthesize and accumulate sulfur- and selenium-containing organic compounds if they have an adequate source of these elements during growth. González-Morales et al. investigate this aspect in their contribution to this special issue [8].

Lastly, returning full circle to allicin, in a paper that will hopefully be useful to allicin researchers worldwide, Albrecht et al. report a facile synthesis and purification procedure to achieve a good yield of highly pure product, based on the already-published favored method of oxidation of diallyl disulfide with a peracid catalyst. Furthermore, they show novel data clarifying the reaction mechanism and kinetics [9].

Thus, this special issue addresses a wide range of contemporary issues relating to the chemistry of alliums as well as the biological effects and potential uses of their organosulfur compounds and the derivatives thereof and will be of interest to students and researchers alike.
